# Postnatal Growth after Intrauterine Growth Restriction Alters Central Leptin Signal and Energy Homeostasis

**DOI:** 10.1371/journal.pone.0030616

**Published:** 2012-01-23

**Authors:** Bérengère Coupé, Isabelle Grit, Philippe Hulin, Gwenaëlle Randuineau, Patricia Parnet

**Affiliations:** 1 UMR 1280 Physiologie des Adaptations Nutritionnelles, Université de Nantes Atlantique, Nantes, France; 2 Research Center of Human Nutrition Nantes, Nantes, France; 3 Plate-forme MicroPICell IFR26 Institut de Recherche Thérapeutique de l'Université de Nantes, Nantes, France; Hôpital Robert Debré, France

## Abstract

Intrauterine growth restriction (IUGR) is closely linked with metabolic diseases, appetite disorders and obesity at adulthood. Leptin, a major adipokine secreted by adipose tissue, circulates in direct proportion to body fat stores, enters the brain and regulates food intake and energy expenditure. Deficient leptin neuronal signalling favours weight gain by affecting central homeostatic circuitry. The aim of this study was to determine if leptin resistance was programmed by perinatal nutritional environment and to decipher potential cellular mechanisms underneath.

We clearly demonstrated that 5 months old IUGR rats develop a decrease of leptin sentivity, characterized by no significant reduction of food intake following an intraperitoneal injection of leptin.

Apart from the resistance to leptin injection, results obtained from IUGR rats submitted to rapid catch-up growth differed from those of IUGR rats with no catch-up since we observed, for the first group only, fat accumulation, increased appetite for food rich in fat and increased leptin synthesis. Centrally, the leptin resistant state of both groups was associated with a complex and not always similar changes in leptin receptor signalling steps. Leptin resistance in IUGR rats submitted to rapid catch-up was associated with alteration in AKT and mTOR pathways. Alternatively, in IUGR rats with no catch-up, leptin resistance was associated with low hypothalamic expression of LepRa and LepRb. This study reveals leptin resistance as an early marker of metabolic disorders that appears before any evidence of body weight increase in IUGR rats but whose mechanisms could depend of nutritional environment of the perinatal period.

## Introduction

Body weight is normally maintained within a narrow range by an appropriate balance between energy intake and energy expenditure. An increase of energy intake leads to excess energy storage in white adipose tissue and weight gain. Genetic background, excessive food consumption, sedentary lifestyle, and decreased physical activity are the main predisposing factors for alteration of energy balance. However a multitude of perinatal factors can alter the metabolic fate of offspring. We previously demonstrated, on a widely used animal model of nutritional programming, that low birth weight, as a consequence of an intrauterine growth restriction (IUGR), leads to metabolic alterations and feeding behaviour abnormalities when followed by a rapid catch-up growth [1]. In complement to that work we demonstrated that rapid catch-up growth of IUGR rats lead to a reduction of leptin sensitivity at postnatal day 5 and 12 in arcuate nucleus (ARC). Since leptin is a critical neurotrophic factor and seems essential for the normal axonal outgrowth of NPY/AgRP and POMC neurons from the ARC to the PVH that occur during that period [2]–[4], a reduced action will have consequence on the ontogeny of hypothalamic regulatory neuronal pathway of food intake [2]. Independently Desai et al. also observed a reduction of leptin activated STAT3 pathway at PND1 after nursing IUGR pups by *ad libitum* fed dams in order to induce a rapid catch-up growth [5]. These and other findings of the literature reveal the important role of nutrition during the perinatal period in adverse adult health outcomes and permanent changes in energy homeostasis [6]–[8].

Leptin is the primary adipose hormones which is produced in proportion to fat stores and circulates as a 16-kDa protein. Adipocytes size is an important determinant of leptin synthesis, since larger adipocytes contain more leptin than smaller [9]. A lack of leptin signalling due to mutation of leptin (*ob/ob*) or the leptin receptor (*db/db*) in mice and in humans results in an increase of food intake concomitant with a reduction of energy expenditure and leads to severe obesity [10], [11].

Leptin binds to the long form leptin receptor (LepRb) [12] predominantly localized in first order neurons of the arcuate nucleus (ARC) and in the second order neurons of ventromedial (VMH), dorsomedial (DMH) and paraventricular (PVH) hypothalamic nuclei [13]–[15]. Leptin acts on ARC neurons, stimulates both POMC expression and neuronal excitability and inhibits AgRP/NPY expression and AgRP neuronal excitability.

Activation of the LepRb results in the phosphorylation of tyrosine residues on JAK2 and three tyrosines on LepRb (Tyr_985_, Tyr_1077_, and Tyr_1138_) [16]. Phosphorylation of LepRb Tyr_1138_ leads to the phosphorylation, dimerization and nuclear translocation of the signal transducer and activator of transcription (STAT3) which activates the transcription of the suppressor of cytokine signaling-3 (SOCS-3). SOCS-3 then binds to Tyr_985_ of LepRb and inhibits its activity. The tyrosine phosphatase, SHP2, also binds LepRb on Tyr_985_ and activates the MAPK cascade via the extracellular signal-regulated kinase (ERK). Leptin also activates phosphatidylinositol3-kinase (PI3-k) pathway and mammalian target of rapamycin (mTOR) via AKT [17]–[19].

In numerous model of diet induced obesity, a hallmark of leptin resistance is **the** impairment of central leptin signalling mostly in hypothalamic neurons but the mechanisms of leptin resistance remains complex and incompletely understood [18]. Several mechanisms may occur and it is likely that, depending on individual circumstances and types of environment as diet or neonatal exposure, leptin could differentially fail to activate or utilize specific signalling cascade to fulfil its role in energy homeostasis. The main mechanisms of leptin resistance are (i) leptin failure to cross the blood–brain barrier because a down-regulation of leptin transporter (as LepRa or LepRe), (ii) hypothalamic LepRb downregulation or (iii) abnormalities in the leptin receptor signalling pathways, as inhibition of the JAK2–STAT3 pathway, overexpression of SOCS-3 [20] impairment of PI3K-mTOR pathway [19] or more recently of the ERK pathway [10], [11], [21].

In this study we analyzed what are the potential reasons of metabolic abnormalities experienced by IUGR rats. Therefore we tested central leptin sensitivity in adult rats. Correlation with adipose tissue morphology, fat depots accumulation and leptin synthesis were also recorded.

## Materials and Methods

### Ethics statement

Animal maintenance and all experiments were conducted in accordance with the European Communities Council Directive of November, 24th 1986 (86/609/EEC) regarding the care and use of animals for experimental procedures and were approved by the Institut National de la Recherche Agronomique (INRA; Paris, France). INRA animal facility was approved by the French Veterinary Department and was registered under the number A44276 (obtained on 16/06/2008).

### Animals

Three experimental groups of pups were created from fourteen pregnant Sprague-Dawley rat dams, housed individually and fed either a normal protein diet (20% of protein) for 6 of them or an isocaloric low protein diet (8% of protein) for 8 of them. Diets were purchased from Arie Block BV (Woerden, The Netherlands) [22]. At delivery, pups born from restricted mothers R or normally fed mothers (C) were adopted randomly to create three experimental groups: CC (n = 3 dams), RC (n = 3 dams) and RR (n = 3 dams) where the first and second letter refers to maternal diet during gestation and lactation, respectively. At birth (postnatal day 0, PND0), rat pups were weighed, and the litters were equalized to eight male pups per litter. After weaning (PND22) all pups were housed individually and fed a control protein diet containing 20% protein. Then, from the age of 40 days, rats were fed a standard laboratory chow (A04, SAFE, Augy, France) until the end of the experiment.

### Food self-selection

Food preferences were analyzed by self selection between chow, high saccharose and high fat diet (Table 1, UPAE, INRA, Jouy en Josas, France) during a consecutive period of 19 days. 4 months rats (n = 10/11 per group) were housed individually under an inverse night/day cycle: 9h00 am/9h00 pm. After a period of habituation to eat from glass cups, 3 different fresh diets were offered daily in a cup (2v∶1v, powder∶water, to minimise spillage) and weighted every morning before night cycle (between 8h00 am and 8h45 am). The places of the different cups in the home cage were changed every day to avoid a home place selection. Each diet was colored by food colorant to quantify spillage.

**Table 1:** Energy and nutrient composition of experimental diets.

	Chow diet	High Fat diet	High Saccharose
**Proteins** (casein)	16.0	16.0	16.0
**Carbohydrate**			
Cornstarch	30.0	8.0	-
Glucose	38.5	38.5	-
Saccharose	-	-	68.5
**Fat**			
Lard	3.0	25.0	3.0
Corn oil	1.0	1.0	1.0
Soy oil	1.0	1.0	1.0
**Fiber** (Cellulose)	6.0	6.0	6.0
**Vitamin and mineral mix**	4.5	4.5	4.5
Energy (kCal/100 g)	387.0	497.0	387.0

Values are in grams per 100 g of diet.

### Blood collection and biochemical analysis

After a fast of 24 h rats were rapidly euthanized between 09h00 am and 11h00 am by CO_2_ inhalation. Blood was collected in heparinised tubes (Laboratoires Léo SA, St Quentin en Yvelines, France) and centrifuged at 2500 g for 15 min at 4°C. Plasma leptin concentrations were determined with specific ELISA kits following the manufacturer's instructions (Rat leptin ELISA kit, LINCO Research, St. Charles, U.S.A.).

### Adipose tissue

Adipose tissue was dissected on 5 months-old rats from three localizations: visceral, retroperitoneal and epididymal fat. This was done by the same experimenter unaware of rat treatments. Adipose tissue was fixed in 4% phosphate-buffered formalin, and then paraffin embedded. Serial sections (5 µm) were stained with hematoxylin-eosine and examined at ×4 magnification using an Eclipse E400 NIKON light microscope equipped with a video camera (Digital camera DXM 1200F). Six images were taken in three different sections per rats in order to collect six images per rats. Each image was analyzed using ImageJ free software. The areas of the adipocytes were measured in a minimum of 200 cells per animals at ×10 magnification in six sections per rats (n = 3/4) and expressed in µm^2^. A mean value was obtained for each animal from at least six representative sections and the size distribution of adipocytes was plotted for comparison between groups.

### Saline and leptin challenge

At 5 months, rats were fasted for 24 hours and received a single intraperitoneal injection of leptin (1 mg/kg) (n = 4, groups) (Preprotech by tebu-bio, Le Perray en Yvelines) or saline (NaCl 0.9%) (n = 3) just before light off. Immediately after the injection, the food tray was filled and weight as well as the rats at t = 0 (time of injection), t = 0.5, 1, 2, 4, 8 and 24 h. Two weeks after the injection, the same rats were fasted for 24 hours and received a new intraperitoneal injection of leptin (1 mg/kg) or saline just before light off. Ninety mins later rats were rapidly euthanized by CO_2_ inhalation. After removal of the brain from the skull, hypothalamus was dissected, according to Paxino's atlas coordinates: −1.0 mm to −4.5 mm from Bregma and 3 mm in depth [23] on ice tray, snapped frozen in liquid nitrogen and stored at −80°C.

### Western Blot analysis

Hypothalami were lysed and proteins extracted in 0.5 ml of Tris HCl 20 mM pH 7.5 with EDTA 1 mM, MgCl_2_ 5 mM, dithiothreitol 1 mM, Na orthovanadate 2 mM with the addition of protease inhibitor cocktail. Protein concentration was quantified by the BCA protein assay kit (Pierce, Thermo Scientific, Rockford, IL) following the manufacturer's instructions. Fifty µg of hypothalamic protein extract were suspended in sample buffer with 5% 2-mercaptoethanol, and boiled for 5 min. Proteins were separated by electrophoresis and transferred to nitrocellulose membranes. The membranes were then incubated for 1 hour at room temperature with 5% fat-free milk in Tris-buffered saline containing 0.1% of tween 20 (TBS/T). The membranes were then incubated 48 h at 4°C with the primary antibody: rabbit anti-phospho-STAT3 (Tyr 705) (1∶1000), rabbit anti-phospho-mTOR (1∶500), rabbit anti-phospho AKT (1∶1000) (Cell Signaling Technology for all antibodies, Ozyme, St Quentin en Yvelines). After washing in TBS/T, the membranes were incubated for 1 hour at room temperature with secondary antibody conjugated with horseradish peroxidase (goat anti-rabbit, 1∶20000, Jackson ImmunuResearch, Interchim, Montluçon). The immunoblots were revealed using enhanced chemiluminescence reagents (Uptilight, Uptima, Interchim) and the membranes were analysed using G:BOX Chemi XL (Syngene, Ozyme, Saint-Quentin-en-Yvelines). After detection of the phosphorylated protein, membranes were stripped using a Tris pH 6.8 50 mM, 2-mercaptoethanol 100 mM and SDS 2% solution and re-blotted with the rabbit anti-STAT3 (1∶1000), and rabbit anti-mTOR (1∶1000), rabbit anti-AKT (1∶2000) (Cell Signaling Technology for all antibodies, Ozyme, St Quentin en Yvelines). Protein expression was quantified with GeneTools software (Syngene, Division of Synoptics Ltd., Cambridge, England) and expressed as the ratio of the phosphorylated form to the total form of the protein. Blots were systematically analyzed with an actin antibody (anti-mouse anti-β-actin antibody, 1/5000, Sigma-Aldrich, St Louis, MO, USA) to verify if proteins were evenly loaded between samples.

### RNA isolation and Real time RT-PCR

RNA was isolated from snap-frozen hypothalamus using the NucleoSpin® RNA/Protein kit (Macherey-Nagel, EURL, Hoerdt, France). Total RNA was submitted to DNase digestion following the manufacturer's instructions, the quality was checked on agarose gels and the quantity estimated by the 260/280 nm UV absorbance. 1 µg of total RNA was reverse-transcribed into cDNA using Random Primer and Moloney Murine Leukemia Virus (MMLV) reverse transcriptase (Promega, Madison, WI, USA) in a total volume of 25 µL. Real time PCR was performed on 5 µL of a 1∶40 dilution of reverse transcribed reaction and 2.5 µM of both forward and reverse primers, in a final volume of 15 µL, using SYBER green PCR kit (Biorad Laboratories, Hercules, CA, USA) in the iCycler iQ® real-time PCR detection system instrument (Biorad). GAPDH (NM_017008) F: cggcaagttcaacggcacag; R: tccacgacatactcagcacca; Ob-Ra (AF-304191) F: cacaccagagaatgaaaaagttgttt; R: tgtagtggtcatgagatacttcaaagag; Ob-Rb (U60151)

F: cacaccagagaatgaaaaagttgttt; R: atgcttggtaaaaagatgctcaa. Negative control for RT-PCR reactions were performed by omitting MMLV from the reaction mixture. mRNA expression was calculated using the 2^−ΔΔCt^ method after normalization with glyceraldehyde 3 phosphate dehydrogenase (Gapdh) as housekeeping gene [24]. Control group values are used as calibrator. The applicability of the CT method was first validated by determining how the amplification efficiencies of the different transcripts including Gapdh varied with template dilution. These experiments showed that the efficiency of the PCR amplification was the same for all the genes and that the expression of Gapdh was not influenced by the pup growth status.

### Statistical analyses

Analyses were performed using Statview 5.0® (SAS Institute Inc.). Differences among groups were determined by Mann-Whitney test and represented as mean ± s.e.m. Repartition of adipocytes was analyzed using Chi2 test. In all tests, P≤0.05 was considered significant.

## Results

### Body weight

Maternal protein restriction during gestation resulted in foetal growth restriction reflected by a significant lower body weight of R rats at birth (Table 2). 5 months-old RC rats display no more significant difference in body weight compared to CC rats (Table 2). At the opposite, body weight of RR rats remained lower compared to CC and RC rats.

**Table 2:** Body weight and fat mass of males CC, RC and RR rats.

	CC	RC	RR
Body weight at birth, g	7.11±0.12	5.96±0.11**	5.96±0.11**
Body weight at 5 months-old, g	606.20±15.21	552.70±18.22	446.55±9.75$$ ##
Visceral fat, g	4.27±0.58	9.86±0.91**	3.90±0.31#
Epididymal fat, g	9.94±0.64	9.05±0.44	5.14±0.32$ #
Retroperitoneal fat, g	15.79±1.87	20.66±1.22	5.72±0.49$ #

Values are means ± s.e.m. n = 6/10 per group, except at birth n = 23 for C and n = 37 for R rats. C: control, RC: protein restricted during gestation. RR: protein restriction during gestation and lactation.

*P<0.05 and

**P<0.01: CC *vs.* RC;

$ P<0.05 and

$$ P<0.01: CC *vs.* RR;

# P<0.05 and

## P<0.01: RC *vs.* RR.

### Food preferences

**Figure 1:**
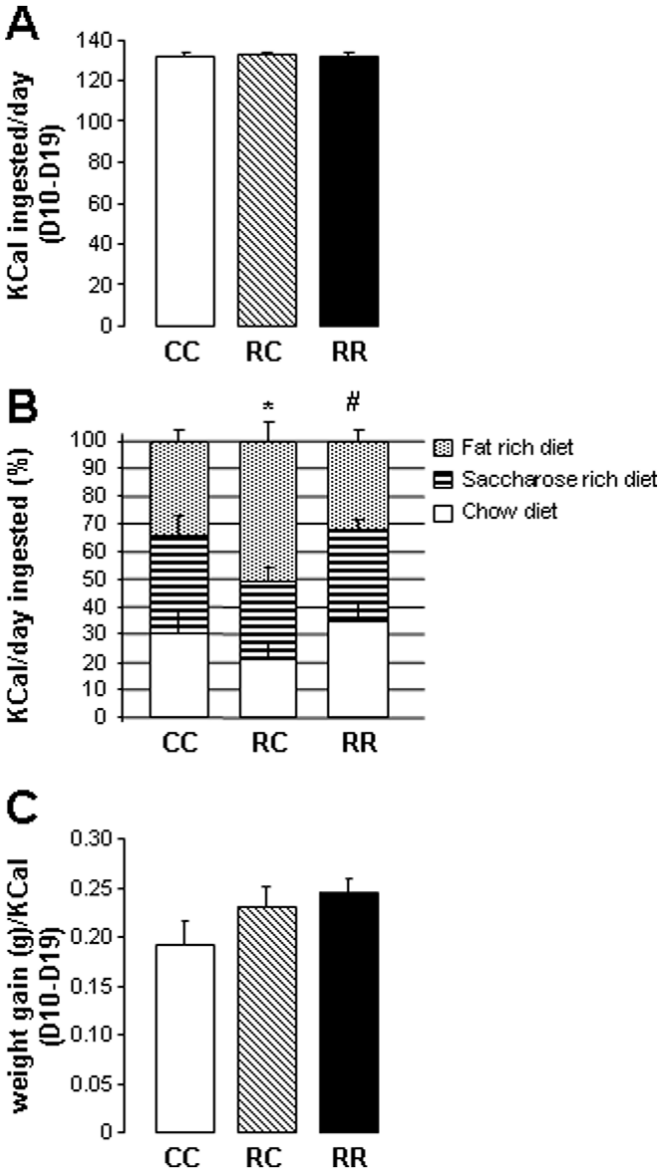
Food consumption analyses demonstrated food preferences among groups at adulthood. (A) Kcal ingested/day, (B) Food preference and (C) Food efficiency during the last 10 days of food self-selection experiment. Values are means ± s.e.m. n = 9/10 per group, *P<0.05: CC *vs.* RC, ^#^P<0.05: RC *vs.* RR.

Data are presented as Kcal ingested/day during the second part (day 10 to day 19) of the self-selection experiment to avoid the analysis of food preferences due to novelty. No difference of Kcal ingested/day was observed between the 3 groups (Fig. 1A). However RC rats demonstrated a different food choice. Indeed, RC rats consumed more high fat diet (50.66±6.62%) and less chow and high saccharose diets, compared to CC and RR rats (34.09±3.75% and. RR: 32.06±3.91%) (Fig. 1B). Additionally food efficiency (weight gain/Kcal ingested) tends to be higher for RC and RR rats although not statistically different when compared to control rats (Fig. 1C).

### Body composition, fat cells size and plasma leptin level

**Figure 2:**
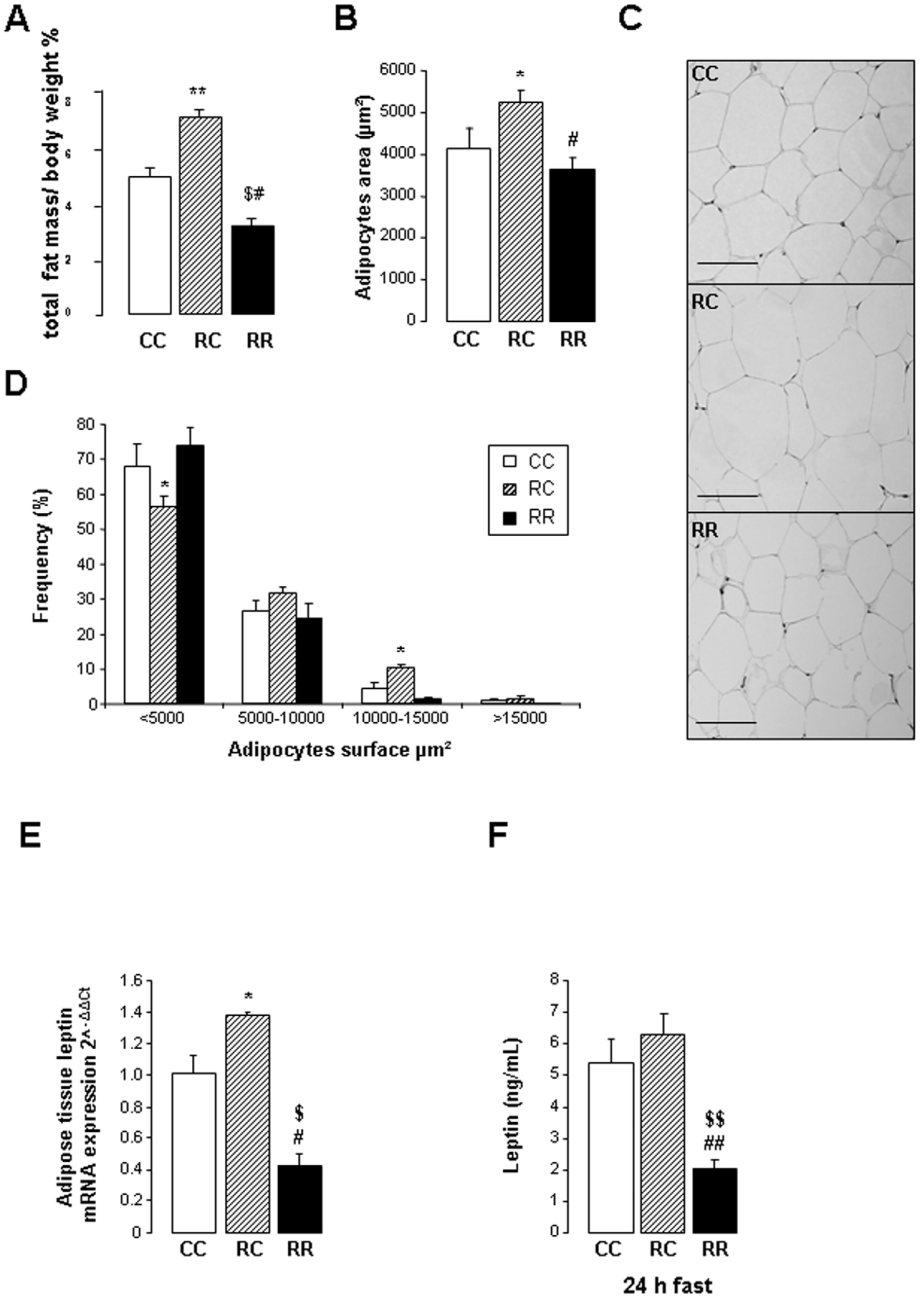
Body composition, fat cells size and plasma leptin level were measured at adulthood. (A) % Total fat mass, (B) adipocytes area (µm^2^), (C) representative photomicrographs of adipose tissue morphology viewed at magnification ×10, Barre scale: 100 µm, (D) adipocytes size repartition evaluated in 5 months-old rats. (E) Relative leptin mRNA expression in adipose tissue, (F) Fast plasma leptin concentrations (ng/ml), Values are means ± s.e.m. *P<0.05: CC *vs.* RC, ^$^P<0.05 and ^$$^P<0.01: CC *vs.* RR, ^#^P<0.05 and ^##^P<0.01: RC *vs.* RR. n = 9/10 per group.

Fat contents were weighted for each group in three different localizations: retroperitoneal, visceral and epididymal (Table 2). Visceral fat deposit was higher in RC rats compared to control rats and although the mass of retroperitoneal fat was not statistically different, they both accounted for a relative adipose tissue weight significantly higher (Fig. 2A). At the opposite fat depots of RR rats was significantly reduced compared to CC and RC rats (Fig. 2A).

The increase of fat depots for RC rats is associated with higher adipocytes surface (Fig. 2B). Considering the adipocytes distribution, RC rats demonstrated a greater number of large adipocytes and a reduction of small adipocytes (≥10 000–15 000 µm^2^, χ^2^ = 120.33, P<0.001) (Fig. 2D). Representative photomicrographs of adipocytes morphology are shown on Figure 2C.

Differences in fat depots (Fig. 2A) and fat cell size (Fig. 2B,C,D) were associated with a significant higher levels of leptin mRNA expression in adipose tissue in RC rats (Fig. 2E) compared to CC rats. Alternatively opposite results were found in RR rats whose fat leptin mRNA expression (Fig. 2E) was associated with a decrease of total fat mass and with lower adipocytes area compared to CC rats (Fig. 2A, B). Plasma leptin levels were correlated to leptin mRNA expression in adipose tissue (Fig. 2F).

### Food intake and body weight after leptin injection

**Figure 3:**
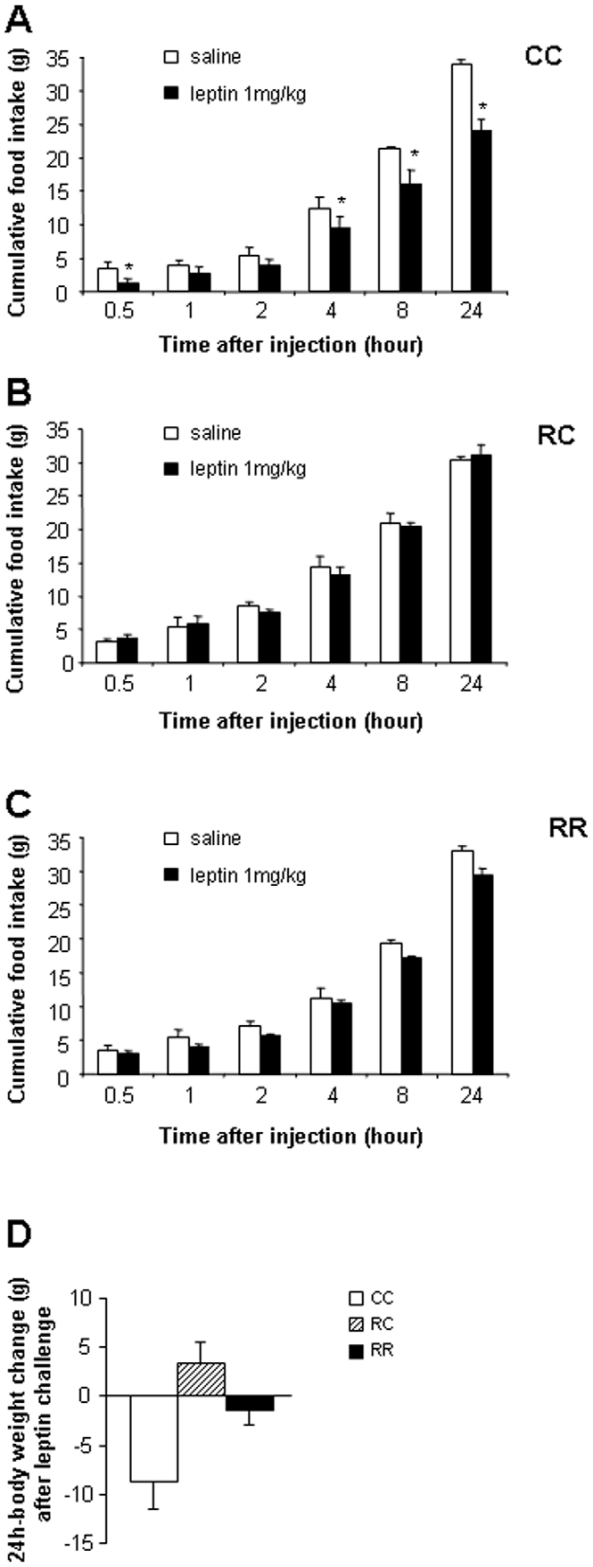
Leptin resistance was evaluated after a 24 h leptin challenge. Food intake (g) measured 0.5, 1,2, 4, 8 and 24 hours after saline or leptin (1 mg/kg) intraperitoneal injections in (A) 5 months-old CC, (B) RC and (C) RR rats. (D) Body weight changes (g) 24 hours after saline or leptin injection. Values are means ± s.e.m; n = 3 per saline treatments; n = 4 per leptin treatment. *P<0.05: saline vs. leptin.

To assess a possible leptin resistance, food intake was measured at 0.5, 1, 2, 4, 8 and 24 hours after saline or leptin (1 mg/kg, i.p) challenge. Cumulative food intake was significantly reduced in CC rats 0.5 h, 4 h, 8 h and 24 h after leptin treatment in comparison to saline injection (Fig. 3A). For RR rats leptin injection induced a lower food intake at each time points that was however not statistically different of saline injected rats (Fig. 3C). RC rats displayed no reduction in food intake after leptin injection therefore the anorexigenic response to the acute dose of peripheral leptin was not observed at all (Fig. 3B). A significant reduction of CC rats body weight was measured 24 hours after leptin injection (Fig. 3D). No reduction of body weight was observed for the RC and RR rats 24 hours after leptin injection (Fig. 3D) suggesting no effect of leptin for RC rats and a diminished effect on RR rats.

### Hypothalamic intracellular pathways activated by leptin challenge

The hypothalamic response was compared in the three groups of fasted rats by quantification of the activation of JAK2/STAT3, AKT and mTOR 90 mins after a single saline or leptin i.p injection. Protein phosphorylation was normalised to total protein. 100% response corresponded to the value of saline treated animals of each group.

**Figure 4:**
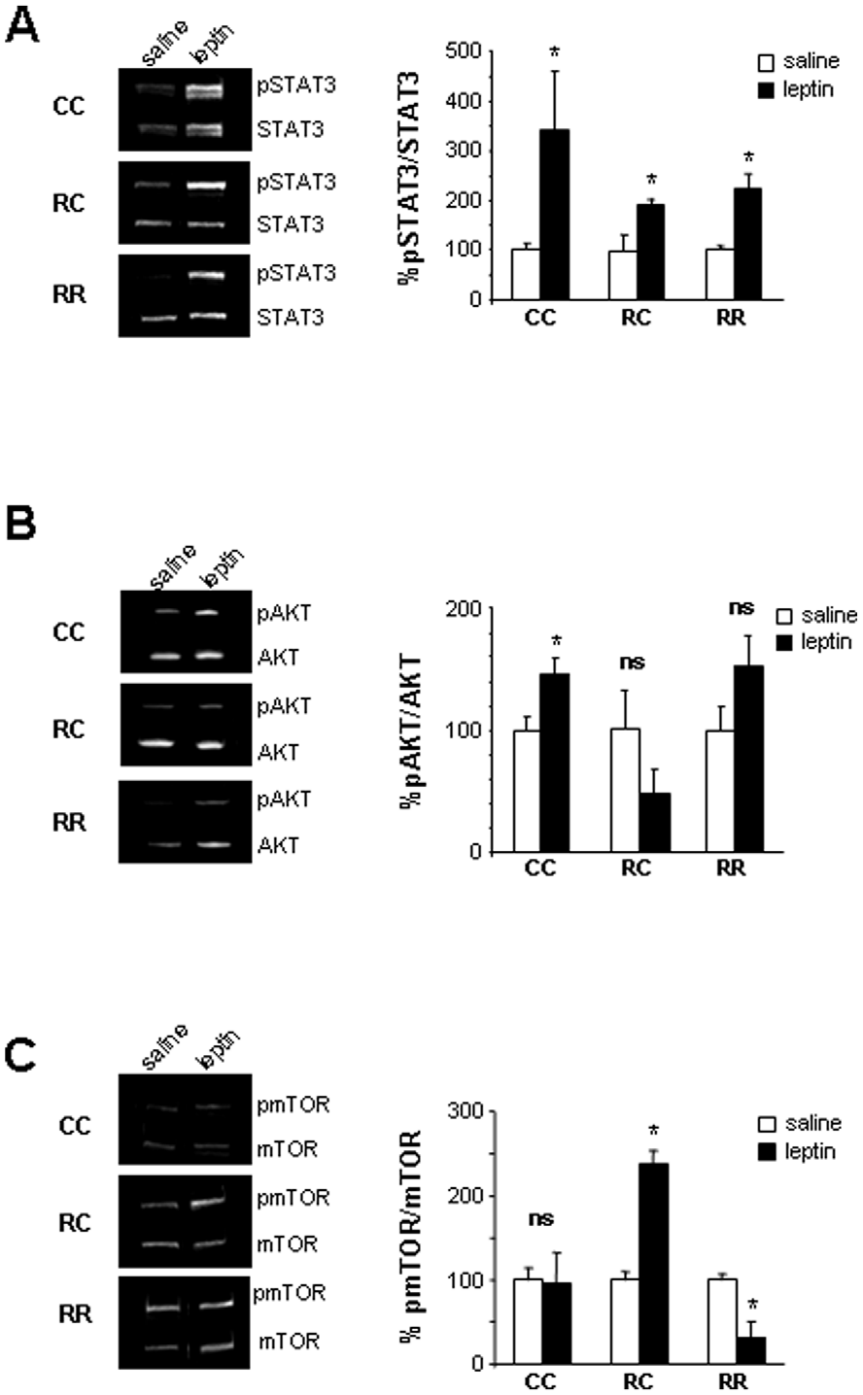
Variations in hypothalamic intracellular pathways activated by leptin challenge. Western blot analysis of (A) phosphorylated and total STAT3, (B) pAKT/AKT, (C) pmTOR/mTOR, in hypothalamic protein extracts from 5 months-old CC, RC and RR rats after saline or leptin (1 mg/kg) intraperitoneal injection. Values are expressed as the ratio of phosphorylated protein/total protein as 100% response represents the value of saline treated animals of each group, n = 3 per saline treatments; n = 4 per leptin treatment. *P<0.05: saline vs. leptin.

Leptin injection induced a significant increase of STAT3 phosphorylation (Fig. 4A). No statistical difference was found in leptin P-STAT3 induction in RC and RR rats compared to CC rats even though RC rats demonstrated a reduced P-STAT3/STAT3 ratio.

Leptin injection induced a significant increase of AKT phosphorylation in CC rats. No leptin activation of this pathway was observed in RC and RR rats (Fig. 4B).

In CC rats, no activation of mTOR pathway was observed after leptin injection. However, RC rats exhibited a significant increase of mTOR phosphorylation and RR rats a significant decrease (Fig. 4C).

### Hypothalamic expression of leptin receptors

**Figure 5:**
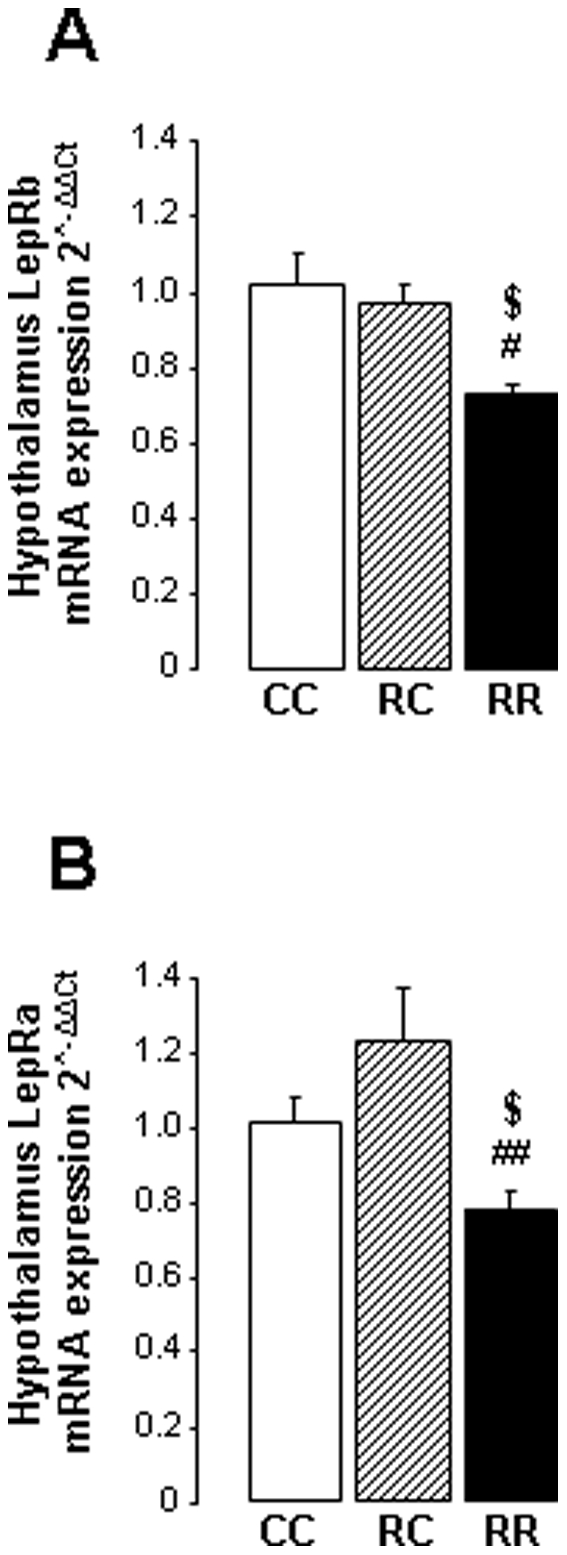
Hypothalamic expression of leptin receptors. (A) Relative hypothalamic LepRb and (B) LepRa mRNA expression. Values are means ± s.e.m. ^$^P<0.05, ^$$^P<0.01: CC *vs.* RR, ^#^P<0.05, ^##^P<0.01: RC *vs.* RR, n = 7–8.

In order to correlate leptin resistance to impaired leptin receptor expression in RC and RR hypothalamus we measured expression of the short form LepRa and the functional long form LepRb receptors. No difference was measured in CC and RC rats for the expression of hypothalamic LepRa and LepRb mRNA. A significant reduction of LepRa (Fig. 5B) and LepRb (Fig. 5B) mRNA was observed in RR hypothalami compared to CC and RC rats.

## Discussion

We previously demonstrated that IUGR leads to food behaviour disorders paired with a strong increase of insulin and leptin secretion after a meal [22]. Since hyperleptinemia is a sign of leptin resistance [25], we hypothesized that an adverse foetal and/or postnatal nutritional environment will program the development of leptin resistance at adulthood.

By the present study we first observed that rapid catch-up growth after IUGR programs the hypertrophy of fat cells and the increase of total fat pads, as soon as 5 months after birth, when animals are not yet overweighed compared to control rats. Then, we demonstrated that leptin challenge failed to reduce appetite of 5 months IUGR rats. Finally we detected anomalies in hypothalamic cellular leptin signals and receptors that could sustain the observed leptin resistance.

In human, rapid catch-up growth of low birth weight babies may increase their risk to develop obesity at adulthood [26]–[28]. With the use of a now classic animal model of IUGR we demonstrated that rapid catch-up growth after IUGR increase the total fat mass of rats that are fed with standard equilibrated rodent chow. While 5 months old IUGR rats did not yet demonstrate obvious sign of obesity they accumulated higher fat deposits and showed a hypertrophy of the adipocytes. In human and animal models metabolic risks and obesity are correlated with a larger visceral adipose tissue [29]. Similar observation were published on a mouse model of catch-up growth which developed an exacerbated adipose tissue at adulthood that even increased when the animals were fed with a high fat diet [30]. Although the RR groups displayed smaller fat pads compared to RC and control group, a previous work of a team of our laboratory showed that they developed higher abdominal fat and a higher increase in serum triglycerides and free fatty levels after exposure to high fat diet than control offspring [31]. Under control chow diet fat mass hypertrophy of the IUGR rats with catch-up growth was associated with a higher expression of leptin mRNA. Additionally in that IUGR rat model we demonstrated plasmatic hyperleptinemia measured shortly after a refeeding period and after high caloric diet intake [1], [22], [30].

Both groups of adult IUGR rats demonstrated an impaired response to leptin challenge since a single peripheral leptin injection did not decrease their food intake nor reduce their weight gain on a 24 h period compared to control rats.

Detection of leptin-stimulated pSTAT3 in the hypothalamus by immunoblotting is an other way to evaluate leptin sensitivity level measurement in hypothalamus [32]. However leptin action in the hypothalamus also mediates signalling by STAT5, ERK, PI3 kinase, mTOR, AMPK and potentially other pathways that are completely or partially independent of STAT-3. Additionally they can be influenced by other factors as insulin or amino acid availability which therefore confounds their use as readouts off cellular leptin signalling. Altough it is known that analysis of these pathways are more difficult to detect than pSTAT3, they may be affected in certain metabolic state and deserve examination [32].

We demonstrated that this leptin resistant state was associated with no significant reduction of hypothalamic pSTAT3 activity but with impaired activation of the PI3K/AKT pathway and a hyper stimulation of mTOR pathway.

A large body of evidence suggests that leptin signalling through STAT3 is critical for maintaining normal energy homeostasis. However in experimental animals as diet-induced obesity (DIO) rats and mice although the anorectic effect of central leptin is reduced, the leptin induced STAT3 activation remained intact for 4 to 19 weeks and becomes impaired after the development of DIO and probably contributes to the maintenance of DIO on a high fat diet [33]. Although disruption of the STAT3 binding site in LepRb or deletion of neuronal STAT3 results in severe hyperphagia and morbid obesity, deletion of STAT3 in either POMC or AgRP neurons only slightly increases food intake and adiposity in mice [34], [35]. This implies that other cellular pathway participate to leptin resistance. It has been demonstrated that hypothalamic PI3K pathway of leptin signalling was impaired in DIO mice fed a high fat diet [33]. Similarly Cota et al, [19], [36] and Martin et al, [37] reported alteration in AMPK and mTOR pathway but not the STAT3 pathway during development of DIO in FVB/N mice. Therefore in absence of significant difference in hypothalamic leptin induced STAT3 activation in RC and RR rats compared to CC rats, we investigated others cellular pathways. The absence of activation and furthermore the inhibition of pAKT signal measured in RC rats could suggest a role of this pathway in leptin resistance since leptin-stimulated activation of hypothalamic PI3-kinase/AKT pathway is impaired in DIO mice [33]. Furthermore inhibition of the PI3-kinase pathway in the brain blocks the ability of leptin to reduce food intake and weight gain.

Leptin stimulates phosphorylation of ribosomal S6 kinase (S6K), a major physiological substrate for mTOR kinase in the hypothalamus [19] but fasting inhibits it. Inhibition of mTOR by rapamycin or deletion of S6K1 attenuates leptin acute anorexigenic action. The high increase of hypothalamic mTOR phosphorylation that we found in RC rats after leptin challenge is not easy to explain in the context of leptin resistance. However in mice, chronic activation of the mTOR/S6K pathway by POMC neuron-specific deletion of TSC1, demonstrate leptin resistance, hyperphagia and obesity, presumably due to an alteration of the hypothalamic neurocircuitry of energy balance [38]. The strong pmTOR signal measured in RC rats could be a major mechanism of leptin resistance but this finding will deserve a better immunohistochemical and anatomical localization in order to phenotype the target cells as well as comparison between the fed and fast state.

In addition to alteration of signalling pathways RR rats hypothalami displayed a significant reduction of the long form leptin receptor (LepRb) mRNA and leptin transporter (LepRa) mRNA. This could result in an impaired response to leptin challenge by leptin failure to cross the blood–brain barrier and low cerebral binding capacity.

In summary, we observed that IUGR rats, with programmed adipocytes hypertrophy by rapid catch-up growth, were leptin resistant prior to the development of obesity. This leptin resistance could involve low activation of the JAK2/STAT3 hypothalamic pathway, deregulation of Akt/mTOR pathway or leptin receptors availability. Leptin resistance represents an early marker of metabolic disorders whose mechanisms could depend of nutritional environment of the perinatal period.
